# MicroRNA-513c-5p is involved in the pathogenesis of preeclampsia by regulating of low-density lipoprotein receptor-associated protein 6

**DOI:** 10.1186/s12884-021-04069-w

**Published:** 2021-12-20

**Authors:** Qian Zhou, Hongyan Li, Yan Zhang, Wei Peng, Haiyan Hou, Mengqi Gu, Fengyuan Zhang, Xietong Wang, Xiao Gu, Lei Li

**Affiliations:** 1 Department of Obstetrics, Shandong Provincial Hospital Affiliated to Shandong First Medical University, 324 Jingwu Road, Jinan, 250021 China; 2 Key Laboratory of Birth Regulation and Control Technology of National Health and Family Planning Commission of China, Maternal Child Health Hospital of Shandong Province, 328 Jingshi East Road, Jinan, China; 3 Department of Obstetrics, The First Affiliated Hospital of Chongqing Medical University, Chongqing, China; 4 Department of Obstetrics, Maternal Child Health Care Hospital of Shandong Province, Jinan, 250014 China; 5 Department of Obstetrics, Shandong Provincial Hospital Affiliated to Shandong University, Jinan, China

**Keywords:** miRNA, Preeclampsia, Trophoblast, miR-513c-5p, Placenta

## Abstract

**Background:**

Preeclampsia (PE) is a major cause of maternal and perinatal morbidity and mortality. Studies on the role of microRNAs (miRNAs), in the pathogenesis of PE through their effects on trophoblast function have been reported, but roles for some miRNAs including miR-513c-5p, have not been identified. We aimed to evaluate potential miRNA candidates that regulate the LRP6 mRNAand to elucidate the possible mechanism in PE. Potential miRNAs were selected by bioinformatics analysis, PCR of placenta tissues and dual luciferase reporter assay of HTR-8/SVneo cells.

**Methods:**

A bioinformatics analysis (Gene Expression Omnibus, GEO; miRWalk) was performed to screen the possible miRNAs that participate in the pathology of PE. Placentas from patients with PE and women with a normal pregnancy were collected to detect the expression of predicted miRNAs by RT-qPCR. A dual luciferase reporter assay was used to test the binding of the potential miRNAs to LRP6. The effects of miR-513c-5p on the biological functions of HTR-8/SVneo cells were further evaluated by performing EdU staining, flow cytometry, wound healing assays and Transwell assays.

**Results:**

GEO and miRWalk predicted 16 miRNAs that might target LRP6. Hsa-miR-371a-5p, hsa-miR-513c-5p, hsa-miR-126-3p, hsa-miR-145-5p, hsa-miR-193b-5p and hsa-miR-296-5p were 6 miRNAs upregulated in the PE placenta. LRP6 was downregulated in patients with PE compared to normal women. miR-513c-5p mimics inhibited LRP6 expression in HTR-8/SVneo cells, and LRP6 is the target gene of miR-513c-5p. miR-513c-5p mimics also inhibited invasion, migration and proliferation of HTR-8/SVneo cells but promoted their apoptosis.

**Conclusions:**

Our study reveals that overexpression of placenta miR-513c-5p is involved in PE by regulating the biological functions of trophoblasts through the inhibition of LRP6.

## Background

Preeclampsia (PE) is defined as hypertension (≥140/90 mmHg) developing after 20 weeks’ gestation with one or more of the following: proteinuria, maternal organ dysfunction (including renal, hepatic, hematological, or neurological complications), or fetal growth restriction [1–4]. The main causes of PE remain unclarified, although it is a major cause causing perinatal morbidity and mortality worldwide [5]. Insights into the pathobiology and diagnosis of PE are lacking. Delivery is considered the only cure, indicating that the placenta is of vital importance in the pathogenesis of PE [6]. The search for novel and more effective biomarkers is expected [7].

MicroRNAs (miRNAs) are small noncoding RNAs, usually 22–24 nucleotides long, which bind to messenger RNAs and repress protein expression. More than 2500 mature human miRNAs exist and have important biological functions [8]. Dysregulation of many miRNAs has been reported to play important roles in many diseases, implying that altered circulating miRNA could serve as potential diagnostic and prognostic biomarkers [9, 10].

Low-density lipoprotein receptor-associated protein 6 (LRP6) is an important coreceptor of the Wnt/β-catenin signaling pathway. As shown in our previous study, LRP6 knockdown in the trophoblast cell line HTR-8/SVneo significantly impairs migration, invasion, and tube formation likely mediated by suppressing Wnt/β-catenin signaling pathway [11]. We further revealed that LRP6 regulates Rab7-mediated autophagy through the Wnt/β-catenin pathway to modulate trophoblast cell migration and invasion [12].

According to our previous studies, LRP6 downregulation potentially contributes to PE development. We aimed to evaluate potential miRNA candidates that regulate the LRP6 mRNA and to elucidate the possible mechanism in PE. In vitro experiments were carried out in this study to elucidate the possible mechanism. Potential miRNAs were seleted by bioinformatics analysis (Gene Expression Omnibus database, GEO, RRID:SCR_005012; miRwalk version 2.0, https://zmf.umm.uni-heideberg.de/apps/zmf/mirwalk2, RRID:SCR_016509), RT-qPCR of placenta tissues and dual luciferase reporter assay of HTR-8/SVneo cells to quantify their levels and make sure the regulation of LRP6 and miRNAs.

## Materials and methods

### Bioinformatics analysis

We selected the GSE15789, GSE69452, GSE84260 and GSE85926 datasets from the GEO, database including patients with PE and normal controls (NCs). Then, an adjusted log2fold change≥1.5 was used as the threshold to identify upregulated miRNAs [13, 14]. miRwalk version 2.0 is a comprehensive database that provides information on predicted miRNAs, as well as the verified binding sites on the target gene. It is currently recognized as miRNA target gene prediction software with a low false-positive rate. “LRP6” was input into the “Gene Targets” frame, select “Human” was selected as the species and 3 ‘UTR as the combined region, and all the prediction software included, this package was used to select miRNAs with frequency ≥ 3/5 [15] and identify miRNAs that may be structurally related to the regulation of LRP6. Next, the GEO and miRWalk results were intersected to identify the candidate miRNAs.

### Dual luciferase reporter assay

A dual luciferase reporter assay was utilized to confirm whether the predicted miRNAs bond to the 3’UTR of LRP6. HEK293T cells purchased from American Type Culture Collection (ATCC, RRID:CVCL_0063) were transfected using X-tremegene HP Transfection Reagent (Roche, Basel, Switzerland,), according to the manufacturer’s protocol. Cells (1.0× 10^5^ cells/well) were seeded in 24-well plates and transfection was performed when HEK293T cells reached 60% confluence in a 24-well plate. Plasmids (GeneChem, Shanghai, China) were mixed in Opti-MEM (Gibco, Burlington, Canada) and incubated with the transfection reagent for 20 min at room temperature. Then the cells were cultured in a 5% CO_2_ incubator at 37 °C for 5 to 6 hours. Twenty-four hours after transfection, the expression of fluorescently labeled genes was observed to determine the transfection efficiency. Forty-eight hours later, luminescence was detected using the Dual-Glo™ Luciferase Assay System (Promega, Madison, USA) according to the manufacturer’s protocol. Data were normalized to Renilla luminescence, and the results are presented relative to the control miRNA transfected group.

### Tissue samples

Twelve pregnant women were included in this study, consisting of 6 patients with PE and 6 women with a normal pregnancy. The study was exploratory and no sample size calculation was performed a priori to assess statistical power. The study was approved by the Institutional Research Ethics Committee of Shandong Provincial Hospital Affiliated to Shandong First Medical University (NO.2019–238), and informed consent forms were signed by every donor. Blood pressure (≥140/90 mmHg) and proteinuria occurring after 34-gestationalweeks were the necessary inclusion criteria for patients with PE, all the patients had headaches and only 2 had fetal growth restriction. Patients who had systemic illnesses were excluded. All donors were delivered by cesarean section and were ≤ 35 years old. Villus tissues form placentas were obtained, placed in TRIzol reagent (Invitrogen, Carlsbad, USA, RRID:Addgene_27409), and stored at − 80 °C until RNA extraction and verification of the differentially expressed miRNAs predicted by the bioinformatics analysis.

### Cell culture and miRNA transfection

The HTR-8/SVneo cell line purchased from ATCC (RRID:CVCL_7162) was maintained in DMEM/F12 (Gibco, Grand Island, USA) supplemented with 10% fetal bovine serum (Gibco, Grand Island, USA), and 1% penicillin and streptomycin in a 5% CO_2_ atmosphere at 37 °C. The miR-513c-5p mimics and negative control were obtained from Gemma Pharma and transfected at a concentration of 50 nM. The two established cell lines sequences were named miR-513c-5p (5′- UUCUCAAGGAGGUGUCGUUUAU-3′) and negative control RNA (NC) (5′- UUCUCCGAACGUGUCACGUTT-3′). Briefly, the transfection reagent, Lipofectamine RNAiMAX (Life Technologies, Carlsbad, USA), was diluted in Opti-MEM and added to the medium; 30 min later, cells were seeded in each well. At 6 h after transfection, the culture medium was replaced with fresh medium; 24 h after plating, cells were harvested for detection. Transfection efficiency was determined by analyzing gene expression 24 h later, using “reverse transcription-quantitative polymerase chain reaction” (RT-qPCR).

### Literature search for miRNAs of interest

PubMed (https://pm.yuntsg.com/) was searched for the needed literature. The name of one of the miRNAs awaiting for dual luciferase reporter assay verification and “trophoblast” were input respectively to retrieve relevant references.

### RNA extraction and RT-qPCR

The quantification and amplification of mRNAs and cDNA quantification in placental tissues and the HTR-8/SVneo cell line were performed using SYBR green-based RT-qPCR, as described previously [16]. TRIzol reagent was used to isolate total RNA from placental tissues or the HTR-8/SVneo cell line. A First Strand cDNA synthesis kit (Thermo Fisher Scientific, Waltham, USA) was utilized to synthesize cDNAs from 1 μg of total RNA. RT-qPCR with gene-specific primers was performed on the resulting cDNAs using Fast SYBR green double-stranded DNA binding dye (Applied Biosystems, Foster city, USA) and a Roche LightCycler® 480 II sequence detection system (Roche, Basel, Swizerland). Primer sequences of LRP6, β-actin, miR-513c-5p and U6 are shown in Table 1. The RT-qPCR profile was as follows: amplification at 37 °C for 15 min, 85 °C for 5 s, and 4 °C before removal. This process was followed by 40 cycles of 95 °C for 10 min, 95 °C for 10 s, 57 °C for 30 s and 72 °C for 10 s. U6 and β-actin were used as internal controls for miRNAs and LRP6, respectively. The experiments were performed in triplicate, and the results were analyzed using the 2^-ΔΔCt^ method.

**Table 1:** Sequences of RT-qPCR primers

LRP6 Forward	TATTGTCCCCCGATGGGCTG
LRP6 Reverse	AGTACATGAACCCACTTGAAGGA
β-actin Forward	TTCCAGCAGATGTGGATCAGC
β-actin Reverse	GAAGCATTTGCGGTGGAC
miR-513c-5p Forward	AGCAGGTTCTCAAGGAGGTGTC
miR-513c-5p Reverse	TAAGGTTCTTCACGACTGGTTCAC
U6 Forward	CAGCACATATACTAAAATTGGAACG
U6 Reverse	ACGAATTTGCGTGTCATCC

### Cell proliferation analysis

The proliferation of HTR-8/SVneo cells was measured using EdU staining (RuiboBio, Guangzhou, China) according to the manufacturer’s protocol. Briefly, HTR-8/SVneo cells were transfected as mentioned above, and 3000 cells were plated in each well of 96-well plates. Twelve hours later, EdU was added to the medium at a final concentration of 50 μM for 1 h. Immunofluorescence staining was performed using EdU reagent kits according to the manufacturer’s instructions. The stained cells were examined with high content screening (Molecular Devices, Silicon Valley, USA) and photographed with a 10× objective. The experiment was performed in triplicate. The positive incidence was proliferative cells/all cells observed, which was defined as red dots/DAPI counts.

### Cell apoptosis analysis

Apoptosis was analyzed using flow cytometry. Twenty-four hours after transfection, HTR-8/SVneo cells were digested with 0.25% trypsin. Apoptotic cells were detected using the Annexin V-FITC/propidium iodide (PI) Apoptosis Detection Kit (Sungene Biotech, Wuhan, China) after treatment. According to the manufacturer’s instructions, the stained cells were assayed using a CytoFLEX flow cytometer (Beckman, Miami, USA) after fixation. The positive cells were calculated and analyzed with CytoExpert 2.0 software (Beckman Coulter, Miami, USA).

### Cell migration analysis

The ability of HTR-8/SVneo cells to migrate was tested by performing wound healing assays. The transfected HTR-8/SVneo cells were cultured in a 96-well plate and stained with PKH67 (Sigma, Saint Louis, USA) for 15 min. The necrotic cells were removed, and then the remaining cells were cultured in serum-free DMEM/F12, which was set as 0 h. The area between the scratches was photographed and calculated by high content screening (10x objective) after 12 h, and the migration of the cells was determined by calculating the scratch area at 0 h - scratch area at 12 h.

### Cell invasion analysis

Transwell assays were performed to assess the invasive ability of HTR-8/SVneo cells. For this experiment. 50 μl of undiluted Matrigel (BD Biosciences, USA) were added to a 8 μm Transwell plate (Merck Millipore, Billerica, USA). After transfection, 5 × 10^5^ HTR-8/SVneo cells were seeded in a Transwell chamber and incubated for 48 h. Then, 4% paraformaldehyde was used to fix the cells in the Transwell chamber, and 0.1% crystal violet was used to stain the HTR-8/SVneo cells on the underside of the Transwell chamber. The invading cells were examined using a digital microscope at 200 x magnification and counted using ImageJ software. Each experiment was repeated three times.

### Statistical analysis

Data is presented as means with standard deviation (Sd). The comparison of demographic characteristics between groups was performed using an unpaired Student’s *t* test. All experiments were of equal variance. All statistical analyses were performed using GraphPad Prism version 5.01. *P* values < 0.05 were considered to be significant (*P < 0.05, **P < 0.01, and ***P < 0.001).

## Results

### Potential miRNAs predicted by bioinformatics analysis

We used the online programs GEO and miRWalk to predict candidate miRNAs that might target LRP6 in humans. Placenta tissue miRNAs of PE and normal pregnant women were search by GEO, and 4 related datasets were obtained. Forty-two upregulated miRNAs were identified (Table 2). miRWalk provided 324 miRNAs according to the set frequency (Table 3). Finally, 16 identified miRNAs that were upregulated in PE and that potentially target LRP6 (hsa-miR-129-2-3p, hsa-miR-409-3p, hsa-miR-765, hsa-miR-371a-5p, hsa-miR-296-5p, hsa-miR-874-3p, hsa-miR-605, hsa-miR-513a-5p, hsa-miR-513c-5p, hsa-miR-126-3p, hsa-miR-143-3p, hsa-miR-145-5p, hsa-miR-193b-5p, hsa-miR-27a-5p, hsa-miR-412-5p, and hsa-miR-497-5p) at the junction of the GEO and miRWalk results.

**Table 2:** 42 upregulated miRNAs filtrated by 4 datasets

Name	logFC
hsa-miR-497-5p	2.506
hsa-miR-199b-5p	2.316
hsa-miR-615-3p	2.301
PREDICTED_MIR192	2.280
hsa-miR-1247-5p	2.193
hsa-miR-455-3p	2.110
PREDICTED_MIR206	2.097
hsa-miR-145-5p	2.066
hsa-miR-513-5p	2.062
hsa-miR-3178	2.042
hsa-miR-455-5p	2.024
hsa-miR-409-3p	1.968
hsa-miR-371-5p	1.950
hsa-miR-199a-5p	1.918
PREDICTED_MIR145	1.888
PREDICTED_MIR172	1.879
PREDICTED_MIR143	1.876
hsa-miR-765	1.871
PREDICTED_MIR112	1.869
hsa-miR-675-5p	1.852
hsa-miR-129-3p	1.831
hsa-miR-27a-5p	1.827
PREDICTED_MIR88	1.814
hsa-miR-1248	1.744
hsa-miR-143-3p	1.731
PREDICTED_MIR160	1.686
hsa-miR-125a-3p	1.665
hsa-miR-199a-3p	1.639
hsa-miR-126-3p	1.637
hsa-miR-668-3p	1.628
hsa-miR-888	1.603
hsa-miR-412-5p	1.596
hsa-miR-6821-5p	1.586
hsa-miR-296-5p	1.582
has-PreMIR-194-2	1.563
hsa-miR-526b-5p	1.557
hsa-miR-10b-5p	1.550
hsa-miR-199b-3p	1.538
hsa-miR-874	1.531
hsa-miR-193b-5p	1.521
hsa-miR-605	1.512
hsa-miR-10a-5p	1.506

A total of 42 upregulated miRNAs(log2 Fold Change≥1.5)obtained by GEO when the 4 sets of miRNAs mentioned were analyzed

**Table 3:** 324 miRNAs predicted by miRWalk

Gene	miRNA	StemLoopID	miRanda	miRDB	miRWalk	RNA22	Targetscan	SUM
LRP6	hsa-miR-183	hsa-mir-183	1	1	1	1	1	5
LRP6	hsa-miR-641	hsa-mir-641	1	1	1	0	1	4
LRP6	hsa-miR-30d	hsa-mir-30d	1	1	1	0	1	4
LRP6	hsa-miR-195	hsa-mir-195	1	1	1	0	1	4
LRP6	hsa-miR-381	hsa-mir-381	1	1	1	0	1	4
LRP6	hsa-miR-424	hsa-mir-424	1	1	1	0	1	4
LRP6	hsa-miR-501-5p	hsa-mir-501	1	1	1	0	1	4
LRP6	hsa-miR-29a	hsa-mir-29a	1	1	1	0	1	4
LRP6	hsa-miR-548i	hsa-mir-548i-4	1	1	1	0	1	4
LRP6	hsa-miR-545	hsa-mir-545	1	1	1	0	1	4
LRP6	hsa-miR-497	hsa-mir-497	1	1	1	0	1	4
LRP6	hsa-miR-450b-5p	hsa-mir-450b	1	1	1	0	1	4
LRP6	hsa-miR-30a	hsa-mir-30a	1	1	1	0	1	4
LRP6	hsa-miR-603	hsa-mir-603	1	1	1	0	1	4
LRP6	hsa-miR-570	hsa-mir-570	1	1	1	0	1	4
LRP6	hsa-miR-30b	hsa-mir-30b	1	1	1	0	1	4
LRP6	hsa-miR-448	hsa-mir-448	1	1	1	0	1	4
LRP6	hsa-miR-624	hsa-mir-624	1	1	1	0	1	4
LRP6	hsa-miR-21	hsa-mir-21	1	1	1	0	1	4
LRP6	hsa-miR-548 h	hsa-mir-548 h-4	1	1	1	0	1	4
LRP6	hsa-miR-454	hsa-mir-454	1	1	1	0	1	4
LRP6	hsa-miR-651	hsa-mir-651	1	1	1	0	1	4
LRP6	hsa-miR-548b-5p	hsa-mir-548b	1	1	1	0	1	4
LRP6	hsa-miR-204	hsa-mir-204	1	1	1	0	1	4
LRP6	hsa-miR-942	hsa-mir-942	1	1	1	0	1	4
LRP6	hsa-miR-559	hsa-mir-559	1	1	1	0	1	4
LRP6	hsa-miR-577	hsa-mir-577	1	1	1	0	1	4
LRP6	hsa-miR-590-5p	hsa-mir-590	1	1	1	0	1	4
LRP6	hsa-miR-205	hsa-mir-205	1	1	1	0	1	4
LRP6	hsa-miR-548c-5p	hsa-mir-548c	1	1	1	0	1	4
LRP6	hsa-miR-15a	hsa-mir-15a	1	1	1	0	1	4
LRP6	hsa-miR-224	hsa-mir-224	1	1	1	0	1	4
LRP6	hsa-miR-579	hsa-mir-579	1	1	1	0	1	4
LRP6	hsa-miR-16	hsa-mir-16-2	1	1	1	0	1	4
LRP6	hsa-miR-548d-5p	hsa-mir-548d-2	1	1	1	0	1	4
LRP6	hsa-miR-548p	hsa-mir-548p	1	1	1	0	1	4
LRP6	hsa-miR-590-3p	hsa-mir-590	1	1	1	0	1	4
LRP6	hsa-miR-211	hsa-mir-211	1	1	1	0	1	4
LRP6	hsa-miR-409-3p	hsa-mir-409	1	0	1	1	1	4
LRP6	hsa-miR-548c-3p	hsa-mir-548c	1	1	1	0	1	4
LRP6	hsa-miR-1270	hsa-mir-1270	1	1	1	0	1	4
LRP6	hsa-miR-580	hsa-mir-580	1	1	1	0	1	4
LRP6	hsa-miR-29c	hsa-mir-29c	1	1	1	0	1	4
LRP6	hsa-miR-29b	hsa-mir-29b-2	1	1	1	0	1	4
LRP6	hsa-miR-15b	hsa-mir-15b	1	1	1	0	1	4
LRP6	hsa-miR-495	hsa-mir-495	1	1	1	0	1	4
LRP6	hsa-miR-300	hsa-mir-300	1	1	1	0	1	4
LRP6	hsa-miR-30c	hsa-mir-30c-1	1	1	1	0	1	4
LRP6	hsa-miR-548a-5p	hsa-mir-548a-3	1	1	1	0	1	4
LRP6	hsa-miR-620	hsa-mir-620	1	1	1	0	1	4
LRP6	hsa-miR-548j	hsa-mir-548j	1	1	1	0	1	4
LRP6	hsa-miR-582-5p	hsa-mir-582	1	1	1	0	1	4
LRP6	hsa-miR-30e	hsa-mir-30e	1	1	1	0	1	4
LRP6	hsa-miR-518a-5p	hsa-mir-518a-2	1	0	1	0	1	3
LRP6	hsa-miR-1285	hsa-mir-1285-1	1	0	1	0	1	3
LRP6	hsa-miR-17	hsa-mir-17	1	0	1	0	1	3
LRP6	hsa-miR-23b	hsa-mir-23b	1	0	1	0	1	3
LRP6	hsa-miR-548 h	hsa-mir-548 h-1	1	0	1	0	1	3
LRP6	hsa-miR-193b	hsa-mir-193b	1	0	1	0	1	3
LRP6	hsa-miR-582-3p	hsa-mir-582	1	0	1	0	1	3
LRP6	hsa-miR-509-5p	hsa-mir-509-2	1	0	1	0	1	3
LRP6	hsa-miR-200a	hsa-mir-200a	1	0	1	0	1	3
LRP6	hsa-miR-548f	hsa-mir-548f-1	1	0	1	0	1	3
LRP6	hsa-miR-654-3p	hsa-mir-654	1	0	1	0	1	3
LRP6	hsa-miR-1302	hsa-mir-1302-2	1	0	1	0	1	3
LRP6	hsa-miR-361-3p	hsa-mir-361	1	0	1	0	1	3
LRP6	hsa-miR-520f	hsa-mir-520f	1	0	1	0	1	3
LRP6	hsa-miR-600	hsa-mir-600	1	0	1	0	1	3
LRP6	hsa-miR-543	hsa-mir-543	1	0	1	0	1	3
LRP6	hsa-miR-623	hsa-mir-623	1	0	1	0	1	3
LRP6	hsa-miR-214	hsa-mir-214	1	0	1	0	1	3
LRP6	hsa-miR-1261	hsa-mir-1261	1	0	1	0	1	3
LRP6	hsa-miR-488	hsa-mir-488	1	0	1	0	1	3
LRP6	hsa-miR-569	hsa-mir-569	1	0	1	0	1	3
LRP6	hsa-miR-320c	hsa-mir-320c-1	1	0	1	0	1	3
LRP6	hsa-miR-105	hsa-mir-105-1	1	0	1	0	1	3
LRP6	hsa-miR-126	hsa-mir-126	1	0	1	0	1	3
LRP6	hsa-miR-320d	hsa-mir-320d-1	1	0	1	0	1	3
LRP6	hsa-miR-520c-3p	hsa-mir-520c	1	0	1	0	1	3
LRP6	hsa-miR-518a-3p	hsa-mir-518a-2	1	0	1	0	1	3
LRP6	hsa-miR-645	hsa-mir-645	1	0	1	0	1	3
LRP6	hsa-miR-1285	hsa-mir-1285-2	1	0	1	0	1	3
LRP6	hsa-miR-20a	hsa-mir-20a	1	0	1	0	1	3
LRP6	hsa-miR-27b	hsa-mir-27b	1	0	1	0	1	3
LRP6	hsa-miR-548 h	hsa-mir-548 h-2	1	0	1	0	1	3
LRP6	hsa-miR-584	hsa-mir-584	1	0	1	0	1	3
LRP6	hsa-miR-7	hsa-mir-7-1	1	0	1	0	1	3
LRP6	hsa-miR-346	hsa-mir-346	1	1	0	0	1	3
LRP6	hsa-miR-340	hsa-mir-340	1	0	1	0	1	3
LRP6	hsa-miR-182	hsa-mir-182	1	0	1	0	1	3
LRP6	hsa-miR-548f	hsa-mir-548f-2	1	0	1	0	1	3
LRP6	hsa-miR-20b	hsa-mir-20b	1	0	1	0	1	3
LRP6	hsa-miR-502-5p	hsa-mir-502	1	0	1	0	1	3
LRP6	hsa-miR-655	hsa-mir-655	1	0	1	0	1	3
LRP6	hsa-miR-1302	hsa-mir-1302-2	1	0	1	0	1	3
LRP6	hsa-miR-141	hsa-mir-141	1	0	1	0	1	3
LRP6	hsa-miR-1279	hsa-mir-1279	1	0	1	0	1	3
LRP6	hsa-miR-362-5p	hsa-mir-362	1	0	1	0	1	3
LRP6	hsa-miR-515-5p	hsa-mir-515-2	1	0	1	0	1	3
LRP6	hsa-miR-217	hsa-mir-217	1	0	1	0	1	3
LRP6	hsa-miR-1262	hsa-mir-1262	1	0	1	0	1	3
LRP6	hsa-miR-491-3p	hsa-mir-491	1	0	1	0	1	3
LRP6	hsa-miR-487b	hsa-mir-487b	1	0	1	0	1	3
LRP6	hsa-miR-34b	hsa-mir-34b	1	0	0	1	1	3
LRP6	hsa-miR-1323	hsa-mir-1323	1	0	1	0	1	3
LRP6	hsa-miR-129-3p	hsa-mir-129-2	1	0	1	0	1	3
LRP6	hsa-miR-320c	hsa-mir-320c-2	1	0	1	0	1	3
LRP6	hsa-miR-369-3p	hsa-mir-369	1	0	1	0	1	3
LRP6	hsa-miR-330-3p	hsa-mir-330	1	0	1	0	1	3
LRP6	hsa-miR-520 h	hsa-mir-520 h	1	0	1	0	1	3
LRP6	hsa-miR-513a-3p	hsa-mir-513a-2	1	1	0	0	1	3
LRP6	hsa-miR-646	hsa-mir-646	1	0	1	0	1	3
LRP6	hsa-miR-1287	hsa-mir-1287	1	0	1	0	1	3
LRP6	hsa-miR-548 h	hsa-mir-548 h-3	1	0	1	0	1	3
LRP6	hsa-miR-548a-3p	hsa-mir-548a-1	1	0	1	0	1	3
LRP6	hsa-miR-874	hsa-mir-874	1	0	1	0	1	3
LRP6	hsa-miR-320a	hsa-mir-320a	1	0	1	0	1	3
LRP6	hsa-miR-302a	hsa-mir-302a	1	0	1	0	1	3
LRP6	hsa-miR-548f	hsa-mir-548f-3	1	0	1	0	1	3
LRP6	hsa-miR-503	hsa-mir-503	1	0	1	0	1	3
LRP6	hsa-miR-656	hsa-mir-656	1	0	1	0	1	3
LRP6	hsa-miR-1302	hsa-mir-1302-3	1	0	1	0	1	3
LRP6	hsa-miR-31	hsa-mir-31	1	0	1	0	1	3
LRP6	hsa-miR-143	hsa-mir-143	1	0	1	0	1	3
LRP6	hsa-miR-1274b	hsa-mir-1274b	1	0	1	0	1	3
LRP6	hsa-miR-519c-3p	hsa-mir-519c	1	0	1	0	1	3
LRP6	hsa-miR-605	hsa-mir-605	1	0	1	0	1	3
LRP6	hsa-miR-301b	hsa-mir-301b	1	0	1	0	1	3
LRP6	hsa-miR-1236	hsa-mir-1236	1	0	1	0	1	3
LRP6	hsa-miR-219-5p	hsa-mir-219-1	1	0	1	0	1	3
LRP6	hsa-miR-548n	hsa-mir-548n	1	0	1	0	1	3
LRP6	hsa-miR-146b-3p	hsa-mir-146b	1	0	1	0	1	3
LRP6	hsa-miR-554	hsa-mir-554	1	0	1	0	1	3
LRP6	hsa-miR-573	hsa-mir-573	1	0	1	0	1	3
LRP6	hsa-miR-1271	hsa-mir-1271	1	0	1	0	1	3
LRP6	hsa-miR-105	hsa-mir-105-2	1	0	1	0	1	3
LRP6	hsa-miR-320d	hsa-mir-320d-2	1	0	1	0	1	3
LRP6	hsa-miR-371-5p	hsa-mir-371	1	0	1	0	1	3
LRP6	hsa-miR-518c	hsa-mir-518c	1	0	1	0	1	3
LRP6	hsa-miR-328	hsa-mir-328	1	0	1	0	1	3
LRP6	hsa-miR-522	hsa-mir-522	1	0	1	0	1	3
LRP6	hsa-miR-650	hsa-mir-650	1	0	1	0	1	3
LRP6	hsa-miR-1290	hsa-mir-1290	1	0	1	0	1	3
LRP6	hsa-miR-124	hsa-mir-124-1	1	0	1	0	1	3
LRP6	hsa-miR-181d	hsa-mir-181d	1	0	1	0	1	3
LRP6	hsa-miR-587	hsa-mir-587	1	0	1	0	1	3
LRP6	hsa-miR-7	hsa-mir-7-2	1	0	1	0	1	3
LRP6	hsa-miR-219-5p	hsa-mir-219-2	1	0	1	0	1	3
LRP6	hsa-miR-548f	hsa-mir-548f-4	1	0	1	0	1	3
LRP6	hsa-miR-505	hsa-mir-505	1	0	1	0	1	3
LRP6	hsa-miR-659	hsa-mir-659	1	0	1	0	1	3
LRP6	hsa-miR-1302	hsa-mir-1302-4	1	0	1	0	1	3
LRP6	hsa-miR-145	hsa-mir-145	1	0	1	0	1	3
LRP6	hsa-miR-1288	hsa-mir-1288	1	0	1	0	1	3
LRP6	hsa-miR-607	hsa-mir-607	1	0	1	0	1	3
LRP6	hsa-miR-509-3-5p	hsa-mir-509-3	1	0	1	0	1	3
LRP6	hsa-miR-1237	hsa-mir-1237	1	0	1	0	1	3
LRP6	hsa-let-7d	hsa-let-7d	1	0	1	0	1	3
LRP6	hsa-miR-221	hsa-mir-221	1	0	1	0	1	3
LRP6	hsa-miR-548 m	hsa-mir-548 m	1	0	1	0	1	3
LRP6	hsa-miR-555	hsa-mir-555	1	0	1	0	1	3
LRP6	hsa-miR-576-5p	hsa-mir-576	1	0	1	0	1	3
LRP6	hsa-miR-149	hsa-mir-149	1	0	1	0	1	3
LRP6	hsa-miR-1826	hsa-mir-1826	1	0	1	0	1	3
LRP6	hsa-miR-372	hsa-mir-372	1	0	1	0	1	3
LRP6	hsa-miR-519d	hsa-mir-519d	1	0	1	0	1	3
LRP6	hsa-miR-629	hsa-mir-629	1	1	0	0	1	3
LRP6	hsa-miR-323-3p	hsa-mir-323	1	0	1	0	1	3
LRP6	hsa-miR-519a	hsa-mir-519a-1	1	0	1	0	1	3
LRP6	hsa-miR-1291	hsa-mir-1291	1	0	1	0	1	3
LRP6	hsa-miR-124	hsa-mir-124-2	1	0	1	0	1	3
LRP6	hsa-miR-302e	hsa-mir-302e	1	0	1	0	1	3
LRP6	hsa-miR-512-5p	hsa-mir-512-1	1	0	1	0	1	3
LRP6	hsa-miR-889	hsa-mir-889	1	0	1	0	1	3
LRP6	hsa-miR-181b	hsa-mir-181b-2	1	0	1	0	1	3
LRP6	hsa-miR-548f	hsa-mir-548f-5	1	0	1	0	1	3
LRP6	hsa-miR-513a-5p	hsa-mir-513a-1	1	0	1	0	1	3
LRP6	hsa-miR-660	hsa-mir-660	1	0	1	0	1	3
LRP6	hsa-miR-1302	hsa-mir-1302-5	1	0	1	0	1	3
LRP6	hsa-miR-520a-3p	hsa-mir-520a	1	0	1	0	1	3
LRP6	hsa-miR-612	hsa-mir-612	1	0	1	0	1	3
LRP6	hsa-miR-935	hsa-mir-935	1	0	1	0	1	3
LRP6	hsa-miR-628-5p	hsa-mir-628	1	0	1	0	1	3
LRP6	hsa-miR-1238	hsa-mir-1238	1	0	1	0	1	3
LRP6	hsa-miR-222	hsa-mir-222	1	0	1	0	1	3
LRP6	hsa-miR-548o	hsa-mir-548o	1	0	1	0	1	3
LRP6	hsa-miR-202	hsa-mir-202	1	0	1	0	1	3
LRP6	hsa-miR-557	hsa-mir-557	1	0	1	0	1	3
LRP6	hsa-miR-576-3p	hsa-mir-576	1	0	1	0	1	3
LRP6	hsa-miR-320b	hsa-mir-320b-2	1	0	1	0	1	3
LRP6	hsa-miR-106a	hsa-mir-106a	1	0	1	0	1	3
LRP6	hsa-miR-186	hsa-mir-186	1	0	1	0	1	3
LRP6	hsa-miR-1827	hsa-mir-1827	1	0	1	0	1	3
LRP6	hsa-miR-520d-3p	hsa-mir-520d	1	0	1	0	1	3
LRP6	hsa-miR-148b	hsa-mir-148b	1	0	1	0	1	3
LRP6	hsa-miR-527	hsa-mir-527	1	0	1	0	1	3
LRP6	hsa-miR-548d-5p	hsa-mir-548d-1	1	0	1	0	1	3
LRP6	hsa-miR-548 k	hsa-mir-548 k	1	0	1	0	1	3
LRP6	hsa-miR-23a	hsa-mir-23a	1	0	1	0	1	3
LRP6	hsa-miR-124	hsa-mir-124-3	1	0	1	0	1	3
LRP6	hsa-miR-302f	hsa-mir-302f	1	0	1	0	1	3
LRP6	hsa-miR-548a-3p	hsa-mir-548a-2	1	0	1	0	1	3
LRP6	hsa-miR-875-5p	hsa-mir-875	1	0	1	0	1	3
LRP6	hsa-miR-7	hsa-mir-7-3	1	0	1	0	1	3
LRP6	hsa-miR-128	hsa-mir-128-2	1	0	1	0	1	3
LRP6	hsa-miR-452	hsa-mir-452	1	0	1	0	1	3
LRP6	hsa-miR-513a-5p	hsa-mir-513a-2	1	0	1	0	1	3
LRP6	hsa-miR-758	hsa-mir-758	1	0	1	0	1	3
LRP6	hsa-miR-93	hsa-mir-93	1	0	1	0	1	3
LRP6	hsa-miR-152	hsa-mir-152	1	0	1	0	1	3
LRP6	hsa-miR-664	hsa-mir-664	1	0	1	0	1	3
LRP6	hsa-miR-302b	hsa-mir-302b	1	0	1	0	1	3
LRP6	hsa-miR-630	hsa-mir-630	1	0	1	0	1	3
LRP6	hsa-miR-1201	hsa-mir-1201	1	0	1	0	1	3
LRP6	hsa-miR-1266	hsa-mir-1266	1	0	1	0	1	3
LRP6	hsa-miR-802	hsa-mir-802	1	0	1	0	1	3
LRP6	hsa-miR-373	hsa-mir-373	1	0	1	0	1	3
LRP6	hsa-miR-520 g	hsa-mir-520 g	1	0	1	0	1	3
LRP6	hsa-miR-194	hsa-mir-194-2	1	0	1	0	1	3
LRP6	hsa-miR-331-5p	hsa-mir-331	1	0	1	0	1	3
LRP6	hsa-miR-519a	hsa-mir-519a-2	1	0	1	0	1	3
LRP6	hsa-miR-548d-3p	hsa-mir-548d-1	1	0	1	0	1	3
LRP6	hsa-miR-128	hsa-mir-128-1	1	0	1	0	1	3
LRP6	hsa-miR-1277	hsa-mir-1277	1	0	1	0	1	3
LRP6	hsa-miR-512-5p	hsa-mir-512-2	1	0	1	0	1	3
LRP6	hsa-miR-190b	hsa-mir-190b	1	0	1	0	1	3
LRP6	hsa-miR-506	hsa-mir-506	1	0	1	0	1	3
LRP6	hsa-miR-1264	hsa-mir-1264	1	0	1	0	1	3
LRP6	hsa-miR-153	hsa-mir-153-1	1	0	1	0	1	3
LRP6	hsa-miR-519b-3p	hsa-mir-519b	1	0	1	0	1	3
LRP6	hsa-miR-633	hsa-mir-633	1	0	1	0	1	3
LRP6	hsa-miR-1206	hsa-mir-1206	1	0	1	0	1	3
LRP6	hsa-miR-1269	hsa-mir-1269	1	0	1	0	1	3
LRP6	hsa-miR-432	hsa-mir-432	1	0	1	0	1	3
LRP6	hsa-miR-561	hsa-mir-561	1	0	1	0	1	3
LRP6	hsa-miR-765	hsa-mir-765	1	0	1	0	1	3
LRP6	hsa-miR-188-5p	hsa-mir-188	1	0	1	0	1	3
LRP6	hsa-miR-374a	hsa-mir-374a	1	0	1	0	1	3
LRP6	hsa-miR-106b	hsa-mir-106b	1	0	1	0	1	3
LRP6	hsa-miR-338-3p	hsa-mir-338	1	0	1	0	1	3
LRP6	hsa-miR-499-5p	hsa-mir-499	1	0	1	0	1	3
LRP6	hsa-miR-548 l	hsa-mir-548 l	1	0	1	0	1	3
LRP6	hsa-miR-130a	hsa-mir-130a	1	0	1	0	1	3
LRP6	hsa-miR-301a	hsa-mir-301a	1	0	1	0	1	3
LRP6	hsa-miR-885-5p	hsa-mir-885	1	0	1	0	1	3
LRP6	hsa-miR-1256	hsa-mir-1256	1	0	1	0	1	3
LRP6	hsa-miR-507	hsa-mir-507	1	0	1	0	1	3
LRP6	hsa-miR-671-5p	hsa-mir-671	1	0	1	0	1	3
LRP6	hsa-miR-29b	hsa-mir-29b-1	1	0	1	0	1	3
LRP6	hsa-miR-153	hsa-mir-153-2	1	0	1	0	1	3
LRP6	hsa-miR-513b	hsa-mir-513b	1	0	1	0	1	3
LRP6	hsa-miR-302c	hsa-mir-302c	1	0	1	0	1	3
LRP6	hsa-miR-297	hsa-mir-297	1	0	1	0	1	3
LRP6	hsa-miR-635	hsa-mir-635	1	0	1	0	1	3
LRP6	hsa-miR-1208	hsa-mir-1208	1	0	1	0	1	3
LRP6	hsa-miR-494	hsa-mir-494	1	0	1	0	1	3
LRP6	hsa-miR-770-5p	hsa-mir-770	1	0	1	0	1	3
LRP6	hsa-miR-190	hsa-mir-190	1	0	1	0	1	3
LRP6	hsa-miR-377	hsa-mir-377	1	0	1	0	1	3
LRP6	hsa-miR-518a-5p	hsa-mir-518a-1	1	0	1	0	1	3
LRP6	hsa-miR-181a	hsa-mir-181a-2	1	0	1	0	1	3
LRP6	hsa-miR-1302	hsa-mir-1302-6	1	0	1	0	1	3
LRP6	hsa-miR-339-5p	hsa-mir-339	1	0	1	0	1	3
LRP6	hsa-miR-499-3p	hsa-mir-499	1	0	1	0	1	3
LRP6	hsa-miR-548d-3p	hsa-mir-548d-2	1	0	1	0	1	3
LRP6	hsa-miR-1302	hsa-mir-1302-1	1	0	1	0	1	3
LRP6	hsa-miR-132	hsa-mir-132	1	0	1	0	1	3
LRP6	hsa-miR-548i	hsa-mir-548i-1	1	0	1	0	1	3
LRP6	hsa-miR-296-5p	hsa-mir-296	1	0	1	0	1	3
LRP6	hsa-miR-520e	hsa-mir-520e	1	0	1	0	1	3
LRP6	hsa-miR-593	hsa-mir-593	1	0	1	0	1	3
LRP6	hsa-miR-212	hsa-mir-212	1	0	1	0	1	3
LRP6	hsa-miR-1257	hsa-mir-1257	1	0	1	0	1	3
LRP6	hsa-miR-412	hsa-mir-412	1	0	1	0	1	3
LRP6	hsa-miR-509-5p	hsa-mir-509-1	1	0	1	0	1	3
LRP6	hsa-miR-767-5p	hsa-mir-767	1	0	1	0	1	3
LRP6	hsa-miR-513c	hsa-mir-513c	1	0	1	0	1	3
LRP6	hsa-miR-518f	hsa-mir-518f	1	0	1	0	1	3
LRP6	hsa-miR-618	hsa-mir-618	1	0	1	0	1	3
LRP6	hsa-miR-1184	hsa-mir-1184	1	0	1	0	1	3
LRP6	hsa-miR-548e	hsa-mir-548e	1	0	1	0	1	3
LRP6	hsa-miR-16	hsa-mir-16-1	1	0	1	0	1	3
LRP6	hsa-miR-1272	hsa-mir-1272	1	0	1	0	1	3
LRP6	hsa-miR-567	hsa-mir-567	1	0	1	0	1	3
LRP6	hsa-miR-581	hsa-mir-581	1	0	1	0	1	3
LRP6	hsa-miR-193a-3p	hsa-mir-193a	1	0	1	0	1	3
LRP6	hsa-miR-518a-3p	hsa-mir-518a-1	1	0	1	0	1	3
LRP6	hsa-miR-181b	hsa-mir-181b-1	1	0	1	0	1	3
LRP6	hsa-miR-1302	hsa-mir-1302-7	1	0	1	0	1	3
LRP6	hsa-miR-500	hsa-mir-500	1	0	1	0	1	3
LRP6	hsa-miR-1302	hsa-mir-1302-2	1	0	1	0	1	3
LRP6	hsa-miR-27a	hsa-mir-27a	1	0	1	0	1	3
LRP6	hsa-miR-548i	hsa-mir-548i-2	1	0	1	0	1	3
LRP6	hsa-miR-130b	hsa-mir-130b	1	0	1	0	1	3
LRP6	hsa-miR-515-5p	hsa-mir-515-1	1	0	1	0	1	3
LRP6	hsa-miR-1184	hsa-mir-1184	1	0	1	0	1	3
LRP6	hsa-miR-181a	hsa-mir-181a-1	1	0	1	0	1	3
LRP6	hsa-miR-1259	hsa-mir-1259	1	0	1	0	1	3
LRP6	hsa-miR-485-5p	hsa-mir-485	1	0	1	0	1	3
LRP6	hsa-miR-510	hsa-mir-510	1	0	1	0	1	3
LRP6	hsa-miR-767–3p	hsa-mir-767	1	0	1	0	1	3
LRP6	hsa-miR-1197	hsa-mir-1197	1	0	1	0	1	3
LRP6	hsa-miR-302d	hsa-mir-302d	1	0	1	0	1	3
LRP6	hsa-miR-520b	hsa-mir-520b	1	0	1	0	1	3
LRP6	hsa-miR-1274a	hsa-mir-1274a	1	0	1	0	1	3
LRP6	hsa-miR-892a	hsa-mir-892a	1	0	1	0	1	3
LRP6	hsa-miR-30c	hsa-mir-30c-2	1	0	1	0	1	3
LRP6	hsa-miR-194	hsa-mir-194-1	1	0	1	0	1	3
LRP6	hsa-miR-380	hsa-mir-380	1	0	1	0	1	3
LRP6	hsa-miR-518d-3p	hsa-mir-518d	1	0	1	0	1	3
LRP6	hsa-miR-181c	hsa-mir-181c	1	0	1	0	1	3
LRP6	hsa-miR-1302	hsa-mir-1302-8	1	0	1	0	1	3
LRP6	hsa-miR-384	hsa-mir-384	1	0	1	0	1	3
LRP6	hsa-miR-1302	hsa-mir-1302-2	1	0	1	0	1	3
LRP6	hsa-miR-137	hsa-mir-137	1	0	1	0	1	3
LRP6	hsa-miR-548i	hsa-mir-548i-3	1	0	1	0	1	3
LRP6	hsa-miR-548a-3p	hsa-mir-548a-3	1	0	1	0	1	3
LRP6	hsa-miR-873	hsa-mir-873	1	0	1	0	1	3
LRP6	hsa-miR-622	hsa-mir-622	1	0	1	0	1	3
LRP6	hsa-miR-1184	hsa-mir-1184	1	0	1	0	1	3
LRP6	hsa-miR-548 g	hsa-mir-548 g	1	0	1	0	1	3
LRP6	hsa-miR-532-5p	hsa-mir-532	1	0	1	0	1	3
LRP6	hsa-miR-320b	hsa-mir-320b-1	1	0	1	0	1	3
LRP6	hsa-miR-518b	hsa-mir-518b	1	0	1	0	1	3

324 miRNAs were predicted by miRWalk that might involve in LRP6 regulation (frequency ≥ 3/5)

### MiR-513c-5p was selected as the target miRNA

**Fig. 1:**
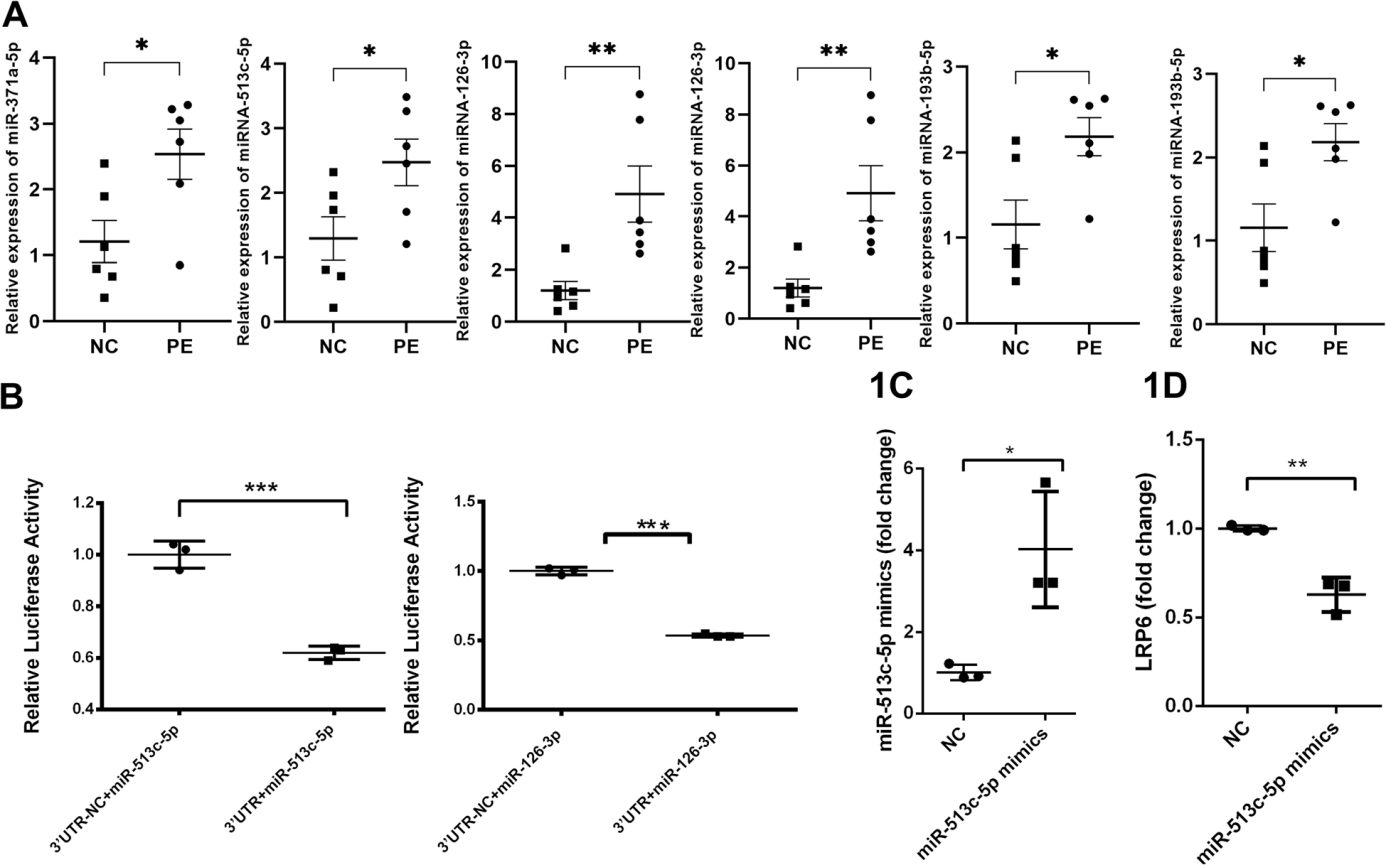
MiR-513c-5p and miR-126-3p targeted the 3’UTR of LRP6. **A** RT-qPCR detected the expression of 16 miRNAs in placental tissues, and 6 miRNAs with increased expression were identified. **B** The 3’UTR of LRP6 harbors one complementary binding site for miR-513c-5p and the miR-126-3p sequences. **C** MiR-513c-5p mimics increased the expression of miR-513c-5p. **D** MiR-513c-5p mimics inhibited LRP6 expression

RT-qPCR of placental tissue from 6 patients with PE and 6 women with normal pregnancies was performed to validate whether the levels of the 16 miRNAs in the placenta were higher in pregnant women with PE than in normal pregnant women (the demographics of the patients are shown in Table 4). Our data revealed that 6 miRNAs (hsa-miR-371a-5p, hsa-miR-513c-5p, hsa-miR-126-3p, hsa-miR-145-5p, hsa-miR-193b-5p and hsa-miR-296-5p) were upregulated and showed a trend toward significance in association with PE (Fig.1a, miR-371a: *P* < 0.05, 1.210 ± 0.714, 2.538 ± 0.853; miR-513c-5p: *P* < 0.05, 1.292 ± 0.754, 2.474 ± 0.809; miR-126-3p: *P* < 0.01, 1.204 ± 0.780, 4.915 ± 2.424; miR-145-5p: *P* < 0.05, 1.116 ± 0.523, 2.060 ± 0.670; miR-193b-5p: *P* < 0.05, 1.170 ± 0.637; miR-296-5p: *P* < 0.05, 1.870 ± 0.602).

**Table 4:** Patient demographics

	PE patients	Normal pregnant women	*P* value
Gestational weeks (minimum-maximum)	35–37	38–40	< 0.000
Systolic blood pressure (mm Hg)	159.2 ± 2.5	108.7 ± 2.1	< 0.000
Diastolic blood pressure (mm Hg)	105.8 ± 3.7	75.3 ± 1.7	< 0.000
Proteinuria(mean ± Sd)	0.771 ± 0.113	0.000 ± 0.000	<0.000
Maternal age	31.830 ± 1.014	29.830 ± 0.792	0.151

Meanwhile, a literature search was needed to better understand the relationship of 6 candidate miRNAs, LPP6 and gestation-related diseases. After a literature search in PubMed, 2 studies at most for each miRNA were acquired. A previous study reported that miR-371a-5p regulates an X-linked inhibitor of apoptosis protein in the pathogenesis of recurrent pregnancy loss [17], and miR-371a-5p promoted the proliferation, migration, and invasion of choriocarcinoma cells [18]. A trend toward the downregulation of miR-126-3p was observed in women with relevant pregnancy-related complications (PE, gestational hypertension and intrauterine growth restriction) compared with women with a normal pregnancy [19, 20]. Tumor necrosis factor-alpha suppresses the invasion of HTR-8/SVneo trophoblast cells through microRNA-145-5p-mediated downregulation of Cyr61 [21]. Higher miR-193b-5p expression in placentae from patients with early-onset pregnancy complications might be involved in the pathogenesis of PE and intrauterine growth restriction [22]. Exosomes containing miR-296-5P have been successfully delivered to recipient cells and might play a biological role in conceptus-endometrial cross-talk crucial for a successful pregnancy [23]. Although the roles of these six miRNAs in the relationship between LRP6 and gestation-related diseases were not explored, five of the six (except miR-513c-5p) mentioned miRNAs were reported to be involved in gestational-related diseases. From a different perspective, the aforementioned literature retrieval process provided evidence for the credibility of GEO and miRWalk analyses, and we wondered whether the candidate miRNAs described above bind to LRP6 and affect trophoblast function.

### Targeting relationship verified by luciferase report assay

Bioinformatics analysis and RT-qPCR selected 6 miRNAs as candidate miRNAs. The miRNA-target relationship was verified by performing a luciferase reporter assay. All 6 miRNAs were identified, but only the binding of miR-513c-5p and miR-126-3p to the targeting sites of LRP6 reduced luciferase expression. The incubation of the 3’UTR plasmids with miR-513c-5p and miR-126-3p resulted in decreased luciferase activity compared to the 3’UTR-NC groups, indicating that miR-513c-5p and miR-126-3p targeted LRP6 (Fig. 1b, miR-513c-5p: *P* < 0.001,1.000 ± 0.053, 0.620 ± 0.027; miR-126-3p:*P* < 0.001, 1.000 ± 0.026,0.537 ± 0.012). Based on these results, miR-513c-5p and miR-126-3p directly target LRP6 and downregulate its expression. In subsequent experiments, miR-513c-5p was randomly selected from the two miRNAs. Further assays of miR-126-3p are still needed.

### MiR-513c-5p regulated the expression of LRP6 in HTR-8/SVneo cells in vitro

The miR-513c-5p mimics and their negative control (NC) were synthesized to further determine the roles of miR-513c-5p in the development of PE. Then, the transfection efficiency of miR-513c-5p mimics in HTR-8/SVneo cells was tested by performing RT-qPCR assay. As presented in Fig. 1c, the introduction of miR-513c-5p mimics detected was higher than the negative control in HTR-8/SVneo cells (*P* < 0.05, 1.011 ± 0.189, 4.022 ± 1.416), suggesting that miR-513c-5p mimics would be useful for subsequent gain-of-function experiments. The LRP6 levels in the miR-513c-5p group and NC group were detected, and the expression of the LRP6 mRNA was reduced in the miR-513c-5p group (Fig. 1d, *P* < 0.01, 1.000 ± 0.0161, 0.627 ± 0.097).

Knockdown of miR-513c-5p inhibited proliferation, invasion migration and promoted apoptosis in HTR-8/SVneo cells.

**Fig. 2:**
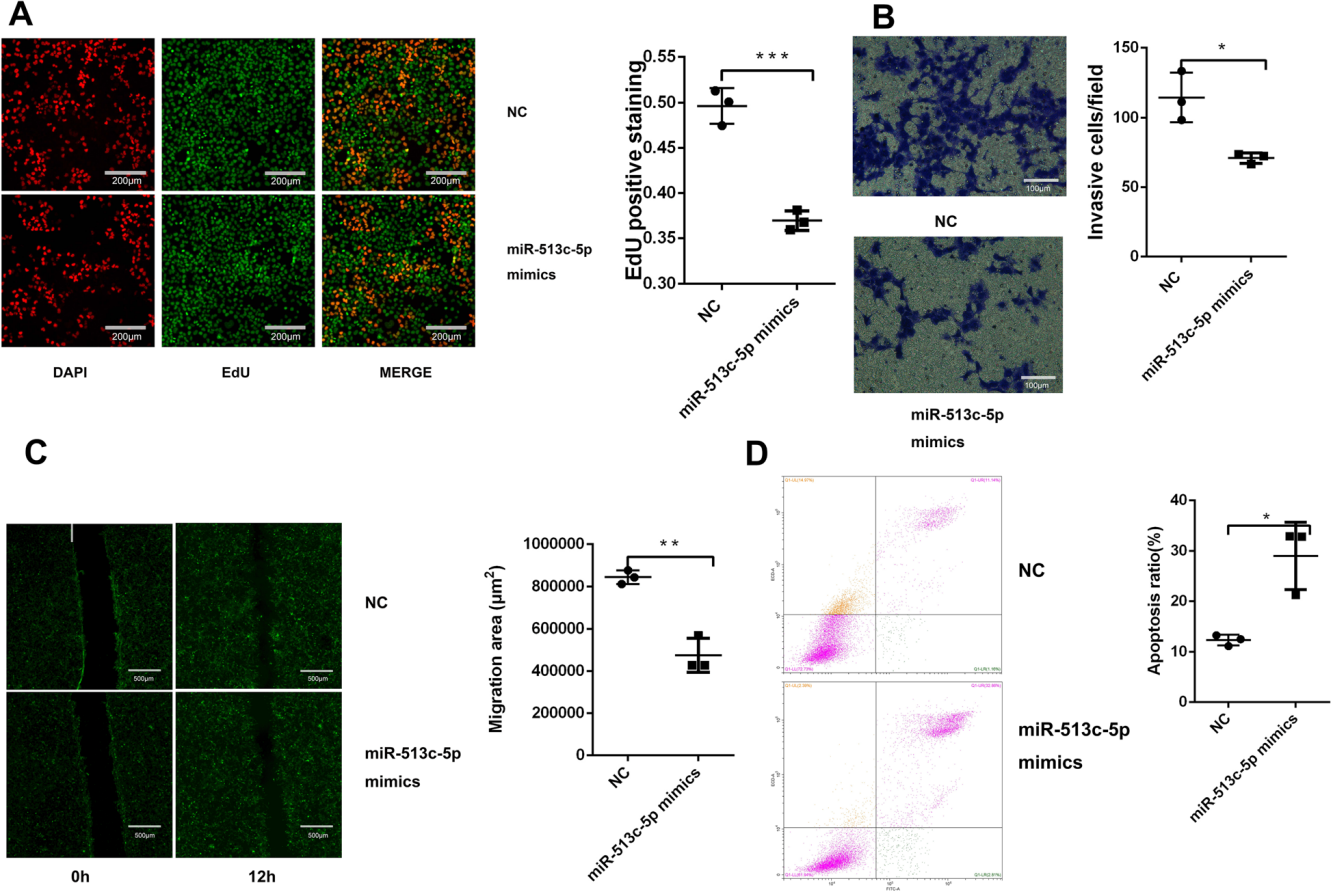
MiR-513c-5p was selected as a miRNA of interest based on the results of the bioinformatic analysis and RT-qPCR. The miR-513c-5p mimic suppressed cell proliferation, invasion and migration but promoted cell apoptosis in HTR-8/SVneo cells. The cell proliferative ability and cell apoptotic rate were determined at 24 h after transfection. **A** The EdU assay showed that the proliferation of HTR-8/SVneo cells was reduced by the miR-513c-5p mimic (*n* = 3). **B** Invasion assays revealed that the invasive ability was inhibited by the miR-513c-5p mimic. The Transwell chamber assay detected the changes in the invasive capabilities of HTR-8/SVneo cells. Compared to NC, trophoblast cells transfected with the miR-513c-5p mimic were significantly less invasive (*P* < 0.05). **C** The miR-513c-5p mimic inhibited the migration of HTR-8/SVneo cells, as analyzed using the wound healing assay. Quantitative analysis of the migration area was assessed. **D** The flow cytometry analysis revealed an increase in the percentage of apoptotic cells after transfection with miR-513c-5p mimics compared with NC

We determined the effect of miR-513c-5p on HTR-8/SVneo cell proliferation using an EdU assay. HTR-8/SVneo cell proliferation was significantly decreased in the miR-513c-5p group compared to the NC group (Fig.2a, *P* < 0.01, 0.496 ± 0.0120, 0.369 ± 0.011). These data indicated that the proliferative ability of trophoblast cells was obviously affected by miR-513c-5p overexpression. Invasion assay depending on Matrigel demonstrated that invasion activity of HTR-8/SVneo cells was remarkably suppressed with excessive miR-513c-5p expression (Fig.2b, *P* < 0.01, 114 ± 18, 71 ± 4). The growth rate of miR-513c-5p overexpressing cells was significantly decreased compared to that of NC cells. Wound healing assays showed that the miR-513c-5p mimic treatment markedly inhibited the motility of HTR-8/SVneo cells, as determined by the migration area (Fig.2c, *P* < 0.01, 843,815 ± 32,623, 474,689 ± 81,106). The cells were analyzed using flow cytometry to determine late apoptotic activity. As shown in Fig.2d, the mimics induced the apoptosis of 28.97 ± 3.87% of HTR-8/SVneo cells, while NC induced the apoptosis of only 12.31 ± 0.62% of cells. Overexpression of miR-513c-5p significantly increased HTR-8/SVneo cells apoptosis (*P* < 0.05, 12 ± 1, 29 ± 7).

## Discussion

Due to the severity of PE, its pathology has always been a research focus. Increasing evidence reveals that miRNAs may participate in the pathology of PE [9, 24, 25]. Accumulating evidence highlights the role of LRP6 in PE. Low LRP6 expression might be responsible for lower trophoblast migration and invasion and subsequent PE, and the mechanisms showed a strong association with Wnt/β-catenin pathway [26]. The overexpression of miR-95-5p regulates the expression of matrix metalloproteinase-2, matrix metalloproteinase-9 and tissue inhibitors of metalloproteinase-1 in trophoblast cells by targeting LRP6, thereby participating in the metastasis of trophoblast cells and causing the occurrence and progression of PE [27]. LRP6 was reported to be involved in the proliferation, migration and invasion of trophoblast cells via miR-346 [28]. Notably, miR-590-3p might inhibit trophoblast-dependent maternal spiral artery remodeling by regulating both trophoblast invasion and endovascular formation through the repression of LRP6 [29]. Based on the results, LRP6 might be regulated by different miRNAs that changing trophoblast function.

In recent years, an increasing number of investigations have regarded miRNAs as participants in the pathogenesis of PE. MiR-513c-5p may have potential value as a cancer marker and has implications for further understanding the molecular basis of different tumor types. MiR-513c-5p is upregulated in breast cancer [30] and is more abundant in sex cord stromal tumors than in ovarian germ cell tumors [31].

In addition, this study also showed that miR-513c-5p expression was negatively associated with the expression of LRP6. A dual luciferase reporter assay indicated that LRP6 was a direct target of miR-513c-5p and that the expression of miR-513c-5p negatively regulated LRP6 expression. Subsequent functional studies showed that miR-513c-5p promoted apoptosis but inhibited the proliferation, invasion and migration of HTR-8/SVneo cells. These results suggested that miR-513c-5p was closely related to the development of PE. Our study partially clarified the role of miR-513c-5p in the development of preeclampsia by regulating LRP6 and provided new suggestions for its specific diagnosis and treatment.

Taken together, miR-513c-5p inhibits trophoblast function by downregulating LRP6. The identification of the inhibitory effects of miR-513c-5p overexpression on trophoblasts may provide insights into potential miRNA-targeted strategies for PE associated with trophoblast dysfunction. According to a recent study, infectious agents, such as human herpesvirus 6, may modulate miRNA expression associated with trophoblast behaviors [32]. Considering the complicated modulatory connections, further studies will be needed to confirm the relationships of miRNAs, trophoblast function and related diseases. Pathological pregnancies were elucidated by trophoblast cell research, and large numbers of studies on the roles of miRNAs in the development of PE have been performed using the HTR8/SVneo cell line due to its efficiency. Because of the drawbacks of cell-based research, for example, the HTR8/SVneo cell line is a mix of trophoblast and stromal/mesenchymal cells [33], further in vivo experiments are needed. In addition, due to the limited sample size of the patients included and differences in gestational age between women with PE and women with normal pregnancies, in vivo experiments are also necessary.

## Conclusions

Our study reveals that overexpression of miR-513c-5p is involved in PE by regulating the biological functions of trophoblasts via inhibition of LRP6.

## Data Availability

All data included in this study are available upon request by contact with the corresponding author.

## Abbreviations

PE: Preeclampsia
miRNA: microRNA
LRP6: Low-density lipoprotein receptor-associated protein 6
GEO: Expression Omnibus
NC: Normal controls
RT-qPCR: Reverse transcription-quantitative polymerase chain reaction
